# Patient satisfaction with pharmaceutical services in Brazilian primary health care

**DOI:** 10.11606/S1518-8787.2017051007145

**Published:** 2017-09-22

**Authors:** Orlando Mario Soeiro, Noêmia Urruth Leão Tavares, José Miguel do Nascimento, Augusto Afonso Guerra, Ediná Alves Costa, Francisco de Assis Acurcio, Ione Aquemi Guibu, Juliana Álvares, Margô Gomes de Oliveira Karnikowski, Silvana Nair Leite, Karen Sarmento Costa

**Affiliations:** I Faculdade de Ciências Farmacêuticas. Pontifícia Universidade Católica de Campinas. Campinas, SP, Brasil; II Departamento de Farmácia. Faculdade de Ciências da Saúde. Universidade de Brasília. Brasília, DF, Brasil; III Prefeitura Municipal de Florianópolis. Florianópolis, SC, Brasil; IV Departamento de Farmácia Social. Faculdade de Farmácia. Universidade Federal de Minas Gerais. Belo Horizonte, MG, Brasil; V Instituto de Saúde Coletiva. Universidade Federal da Bahia. Salvador, BA, Brasil; VI Departamento de Saúde Coletiva. Faculdade de Ciências Médicas da Santa Casa de São Paulo. São Paulo, SP, Brasil; VII Faculdade de Ceilândia. Universidade de Brasília. Brasília, DF, Brasil; VIII Departamento de Ciências Farmacêuticas. Universidade Federal de Santa Catarina. Florianópolis, SC, Brasil; IX Núcleo de Estudos de Políticas Públicas. Universidade Estadual de Campinas. Campinas, SP, Brasil; X Programa de Pós-Graduação em Saúde Coletiva. Departamento de Saúde Coletiva. Faculdade de Ciências Médicas. Universidade Estadual de Campinas. Campinas, SP, Brasil; XI Programa de Pós-Graduação em Epidemiologia. Faculdade de Medicina. Universidade Federal do Rio Grande do Sul. Porto Alegre, RS, Brasil

**Keywords:** Patient Satisfaction, Pharmaceutical Services, Primary Health Care, Health Services Research, Unified Health System, Satisfação do Paciente, Assistência Farmacêutica, Atenção Primária à Saúde, Pesquisa sobre Serviços de Saúde, Sistema Único de Saúde

## Abstract

**OBJECTIVE:**

To evaluate patient satisfaction with pharmaceutical services in Brazilian primary health care.

**METHODS:**

This is a cross-sectional, exploratory, and evaluative study on a representative sample from the five Brazilian geopolitical regions resulting from the *Pesquisa Nacional sobre Acesso, Utilização e Promoção do Uso Racional de Medicamentos – Serviços, 2015* (PNAUM – National Survey on Access, Use and Promotion of Rational Use of Medicines – Services, 2015). The outcome was the patient’s satisfaction, obtained using the item response theory. Associations were tested using Pearson’s Chi-square test with sociodemographic and health variables, and multiple logistic regression analyses were carried out. The Hosmer-Lemeshow test was used to verify the adequacy of the final model. Logistic regression results were presented as odds ratio.

**RESULTS:**

The overall percentage of patients satisfied with these services was 58.4% (95%CI 54.4-62.3). The “opportunity/convenience” aspect had the lowest satisfaction percentage (49.5%; 95%CI 46.4-52.6) and “interpersonal aspects,” the highest percentage (90.5%; 95%CI 88.9-91.8), significantly higher than other aspects. Sex, age group, limitations due to disease, and self-perception of health remained associated in the final multiple logistic model regarding general satisfaction.

**CONCLUSIONS:**

Most of the interviewed users were satisfied with pharmaceutical services in Brazilian cities, and the satisfaction with the customer’s service was determinant in the patient’s overall satisfaction.

## RESUMO

**OBJETIVO:**

Avaliar a satisfação dos usuários com os serviços de assistência farmacêutica na atenção primária.

**MÉTODOS:**

Estudo transversal, exploratório, de natureza avaliativa, com amostra representativa das cinco regiões geopolíticas do Brasil, oriundas da Pesquisa Nacional sobre Acesso, Utilização e Promoção do Uso Racional de Medicamentos – Serviços, 2015. O desfecho foi a satisfação do usuário obtida pelo método de teoria da resposta ao item. Foram testadas associações por meio do teste qui-quadrado de Pearson com variáveis sociodemográficas e de saúde e realizadas análises de regressão logística múltipla. O teste de Hosmer-Lemeshow foi utilizado para verificar a adequação do modelo final. Os resultados da regressão logística foram apresentados por meio de razão de chances.

**RESULTADOS:**

O percentual geral de usuários satisfeitos com esses serviços foi de 58,4% (IC95% 54,4–62,3). A dimensão oportunidade/conveniência apresentou menor percentual de satisfação (49,5%; IC95% 46,4–52,6) e o maior percentual foi na dimensão aspectos interpessoais (90,5%; IC95%88,9–91,8), significativamente maior que as demais dimensões. No modelo logístico múltiplo final em relação à satisfação geral, permaneceram associadas as variáveis sexo, faixa etária, limitação por doenças e autopercepção da saúde.

**CONCLUSÕES:**

A maior parte dos usuários entrevistados mostrou-se satisfeita com os serviços da assistência farmacêutica dos municípios brasileiros e a satisfação com o atendimento apresentou-se como fator relevante na satisfação geral do usuário.

## INTRODUCTION

The evaluation of policies and programs is essential in public health, because it contributes to the efforts towards a healthier society and avoids wasting resources with inefficient programs^9^ ^,^ ^24^. However, when evaluating the effects of a health policy in the services performance and in the general health status of a population, it is necessary to appreciate the synergy between the determinants associated with the health policy, the health services (structures, human resources, and processes), and the state of health and needs of a population^9^ ^,^ ^10^ ^,^ ^19^ ^,^ ^25^.

Since the creation of the Brazilian Unified Health System (SUS), in 1990, different initiatives toward health evaluation have been developed. At first, surveys were intended to evaluate health services and facilities, and they were almost exclusively developed within universities. Later, the same rationalizing policies that valued planning in healthcare became concerned with the issue of evaluation. In the 1980s, through an integrated scheduling and budgeting and the first state and municipal health plans, the *Ações Integradas de Saúde* (AIS – Integrated Health Actions) and the *Sistemas Unificados e Descentralizados de Saúde* (SUDS – Unified and Decentralized Health Systems) also took into account the planning and evaluation^16^.

In the 1990s, the achievement of the institutionalization of community participation in planning and evaluation processes and the strengthening of social regulation within SUS were important factors to the development of evaluation studies, which assume patients are able to evaluate, intervene, and change the system, according to their own needs^12^ ^,^ ^23^.

Despite emphasis given by the new health policies to social regulation in Brazil, listening to the user has not been a common practice in health care. In order for this organizational principle of SUS may be implemented in the everyday activities of health services, along with doctrinal principles of universality, integrality, and equity, the development of researches to analyze, evaluate, and interpret the demands and needs of patients who use these services becomes relevant. To listen to and welcome patients are also ways of ensuring the right to health and citizenship^17^.

Evaluation of health care in public and private institutions is one of the management tools to constantly improve the quality of service provided. It is an intentional, technical, and political process, but also a social and ethical responsibility. The concern with the quality of the professional-patient relationship becomes a means to an end^9^ and an end in itself.

Polysemy in health evaluations is a phenomenon reproduced by many authors in this area. Just as the term “evaluation” has a broad range of meanings, “patient satisfaction” also presents terminological difficulties. Patient satisfaction, according to Linder-Pelz, consists of evaluating different aspects of health care, assigned positively and individually by the patient^15^.

Silva and Formigli^20^ affirm that patient satisfaction is based on “a subjective understanding the individual has on the care received”. Therefore, the degree of satisfaction or dissatisfaction with the health service may refer to the relationship with the health care professional, but also to aspects of service infrastructure (equipment and medicines), amenities (ventilation and comfort), and their representations in the health-disease process^20^.

Studies showed that satisfied patients usually adhere to the treatment prescribed, provide important information to the health care provider, and continue using health services. It is also mentioned that satisfied patients are more likely to have better a quality of life^1^ ^,^ ^4^ ^,^ ^6^ ^,^ ^26^ ^-^ ^28^. In this context, patient satisfaction is considered a goal to be achieved by health care services and, therefore, should be researched to incorporate improvements in the health care system.

The *Pesquisa Nacional sobre Acesso, Utilização e Promoção do Uso Racional de Medicamentos – Serviços* (PNAUM – National Survey on Access, Use and Promotion of Rational Use of Medicines – Services) aimed at characterizing the coordination of pharmaceutical services in the primary health care of SUS, to promote the access and rational use of medicines, as well as to identify and discuss the factors that affect the consolidation of pharmaceutical services in the cities.

This study is part of PNAUM – Services, and aims at evaluating the patient satisfaction with pharmaceutical services in the primary health care of Brazilian cities.

## METHODS

PNAUM is a cross-sectional, exploratory, and evaluative study, consisting of a survey on a representative sample of primary health care services of cities from the Brazilian regions. Several study populations were included in the sampling plan, with samples stratified by Brazilian regions, which comprise the study domains^2^.

In-person interviews were conducted with patients, doctors and professionals responsible for supplying medicines in SUS primary health care services. In addition, conditions of the pharmaceutical services facilities were observed and telephone interviews to professionals responsible for pharmaceutical services in the cities were conducted. Data was collected between July and December 2014.

PNAUM – Services methodology, as well as the sampling process, are described in detail by Álvares et al.^2^ The tool has been tested previously and questions from the Long-Form Patient Satisfaction Questionnaire (PSQ-III), adapted for pharmaceutical services, were used as a reference to create the questions on patient satisfaction^13^.

Analysis of patient satisfaction in its different aspects was carried out considering the total sample of 8,803 patients, interviewed during data collection. The tool was used by previously trained interviewers and the sampling process was carried out based on the medical consultation schedule of SUS units, per day of the week. Names were listed in alphabetical order and interviews made according to alphabetical order. After this stage, the first patient to be interviewed was identified during a medical consultation of any of the doctors, and the respondent would be the last patient, among those who were already in the unit.

To create the analysis of satisfaction the following aspects of access to medicines were considered: availability (AVAIL), opportunity/convenience (OP/CO), and adequacy. Adequacy aspect was assessed in the following sub-aspects: technical quality of dispensation (QDISP), technical quality of the medicine (QMED), ambiance (AMB), and interpersonal aspects (IA)^11^.

The dependent variable of the study was patient satisfaction, obtained using the item response theory (IRT)^3^, considering the variable-answers of six of the evaluated aspects in patient satisfaction: opportunity/convenience, availability, ambiance, interpersonal aspects, quality of medicines, and quality of dispensation. Questions used for each aspect, as well as their categorization are described in Box. Answers 1 indicated satisfaction and answers 0 indicated dissatisfaction.

**Box:** Questions considered in the analysis of patient satisfaction with pharmaceutical services in the primary health care, according to aspects and sub-aspects, and categorization for the item response theory model. National Survey on Access, Use and Promotion of Rational Use of Medicines – Services, 2015.

Questions	Categorization
**Opportunity Aspect**	
Is this place far from your house?	Assume value *1* for the answer “no” and *0* for the answers “yes” and “more or less”.
*Arrive at the Health Care Unit*	“Very easy” and “Easy” received code *1*; the answers “neither easy/nor difficult,” “difficult,” and “very difficult” received code *0*.
*Opening hours of this health care unit*	“Very good” and “good” received code *1*; the answers “neither bad/nor good,” “bad” and “very bad” received code *0*.
*How long do you usually wait to obtain the medicines in public pharmacies of SUS?*	The answers “do not wait” and “a little” received code *1* and the answer “a long time” received *0*.
**Availability Aspect**	
*Where did you get this medicine the last time?*	The answers “Pharmacy of the SUS,” “Popular Pharmacy Program,” or “Church or Union” received code *1* (these answers would be related to an easier access to medicine, promoting satisfaction) and the answer “Commercial Pharmacy” received code *0*.
Did you have any problems to get the medicine the last time?	The answers “did not have problems” received code *1* and the answers that indicated that the patient had problems to get the medicine received code *0*.
*Did you stop taking the medicine for any reasons in the past seven days?*	“No” had value *1* and “yes,” value *0*. In the case of patients who have taken more than one medicine, if there was a medicine they stopped taking, the answer would be *0*.
*In the past three months, how often did you get the medicines you needed in public pharmacies of SUS?*	The answers “always” and “repeatedly” received code *1*; the answers “sometimes,” “rarely,” and “never” received code *0*.
*Did you have problems remembering to take the medicine?*	The answer “no” received code *1* and the answer “yes,” code *0*.
*Did you have problems with the medicines for taking many pills per day?*	
*Did you have problems with the medicines for being hard to obtain?*	
*Did you have problems with the medicines because it is hard to read the packaging?*	
*Did you have problems with the medicines because it is hard to adapt their use with your working routines?*	
*Did you have problems with the medicines because there are different medicines with the same shape and colors?*	
**Quality of dispensation sub-aspect**	
When you obtain the medicines in the public pharmacies of SUS, do the public employees who deliver the medicines give you information about their use?	The answer “yes” received code *1;* the answers “sometimes” and “no,” code *0*.
Do you understand the information given by employees who deliver the medicines in the public pharmacies of SUS?	The answers “always” and “repeatedly” received code *1*; the answers “sometimes,” “rarely,” and “never” received code *0*.
Is the pharmacist or another employee from the public pharmacy of SUS available when you need to ask questions about the medicines?	
Quality of medicines sub-aspect	The answer “well” received code *1*; the answers “regular” and “does not work well” received code *0*. In the case of patients who have taken more than one medicine, if there was a medicine they thought was working neither good nor badly or was not working well, the answer would be *0*.
In your opinion, is this medicine causing you any health problems?	The answer “no” received code *1* and “yes,” code *0*. In the case of patients who have taken more than one medicine, if there was a medicine that caused them any health problems, the answer would be *0*.
For you, the effects of medicines obtained in public pharmacies of SUS compared to the effects of medicines bought in commercial pharmacies are:	“The same” and “better” received code *1* and “worse,” code *0*.
When you obtain medicines in public pharmacies of SUS, do you receive information on how to store them at home?	The answers “always” and “repeatedly” received code *1*; the answers “sometimes,” “rarely,” and “never” received code *0*.
Ambiance Sub-aspect	
*How do you evaluate the wall signs to find the public pharmacy of SUS?*	“Very easy” and “easy” received code *1*, the answers “neither easy/nor difficult,” “difficult,” and “very difficult” received code *0*.
*How do you evaluate the cleanliness of the public pharmacy of SUS?*	The answers “very good” and “good” received code *1* and the answers “neither bad/nor good,” “bad,” and “very bad” received code *0*.
*How do you evaluate the comfort in the public pharmacy of SUS?*	The answers “very good” and “good” received code *1* and the answers “neither bad/nor good,” “bad,” and “very bad” received code *0*.
*How do evaluate the service of the public pharmacy of SUS?*	
**Interpersonal aspects sub-aspect**	
*Is the personnel of the public pharmacy of SUS respectful and polite?*	The answers “always” and “frequently” received code *1*, and the answers “sometimes,” “rarely,” and “never” received code *0*.
Do you consider that the service provided at the pharmacy of SUS respects your privacy?	
*How do you evaluate the service of the public pharmacy of SUS?*	The answers “very good” and “good” received code *1,* and the answers “neither bad/nor good,” “bad,” and “very bad” received code *0*.

SUS: Brazilian Unified Health System

Source: PNAUM – Services, 2015.

The satisfaction rate for the outcome was created, transformed into a scale of 0 to 1, in which values closer to 0 indicated higher dissatisfaction and closer to 1 indicated higher satisfaction. For the variable answer of the analysis, the transformed rate was categorized as less than 0.50 (unhappy) and greater than or equal to 0.50 (satisfied). Data analysis was performed using the software SPSS® version 22, considering a complex sample design^1^. Descriptive analysis was performed presenting absolute and relative frequencies, and confidence intervals for relative frequencies.

The resulting assotiations were tested and categorized through the Pearson’s Chi-square test, with the following independent variables: sex, age range, marital status, skin color, education, economic classification according to the *Associação Brasileira de Empresas de Pesquisa* (ABEP – Brazilian Association of Research Enterprises^a^), region of residence, having health insurance, patient of SUS, number of hospitalizations in the past 12 months, number of emergency services required in the past 12 months, self-perception of health, number of chronic diseases, limitations due to diseases, pain and discomfort, anxiety and depression, and number of medicines used.

Variables with p<0.2 were included in the multiple logistic model, in which only those with p < 0.05 remained. Logistic regression results were displayed as odds ratio with their respective 95%CI. The Hosmer-Lemeshow test was used to assess the adequacy of the final model.

PNAUM was approved by the National Research Ethics Committee of the National Health Council, under Opinion no. 398,131/2013. All participants signed the informed consent form.

## RESULTS

The overall percentage of patients who were satisfied with the pharmaceutical services in primary health care was 58.4% (95%CI 54.4–62.3). When analyzing patient satisfaction in each aspect evaluated, we observed the lowest percentage of satisfaction for the opportunity/convenience aspect (49.5%; 95%CI 46.4–52.6) and the highest for interpersonal aspects (90.5%; 95%CI 88.9–91.8), significantly higher than other aspects. We also observed similar satisfaction rates for opportunity/convenience, availability, and ambiance. The satisfaction rate for the quality of medicines and the quality of dispensation presented similar satisfaction rates (Box).

Regarding the variables concerning opportunity, patients reported higher satisfaction with the units’ opening hours (85.7%) and with waiting time to obtain their medicines (95%). Regarding availability, most patients (65.1%) said they did not have problems the last time they obtained their medicines and 67% obtained the medicines they needed in the Popular Pharmacy Program of SUS during the past three months (Figure).

**Figure:**
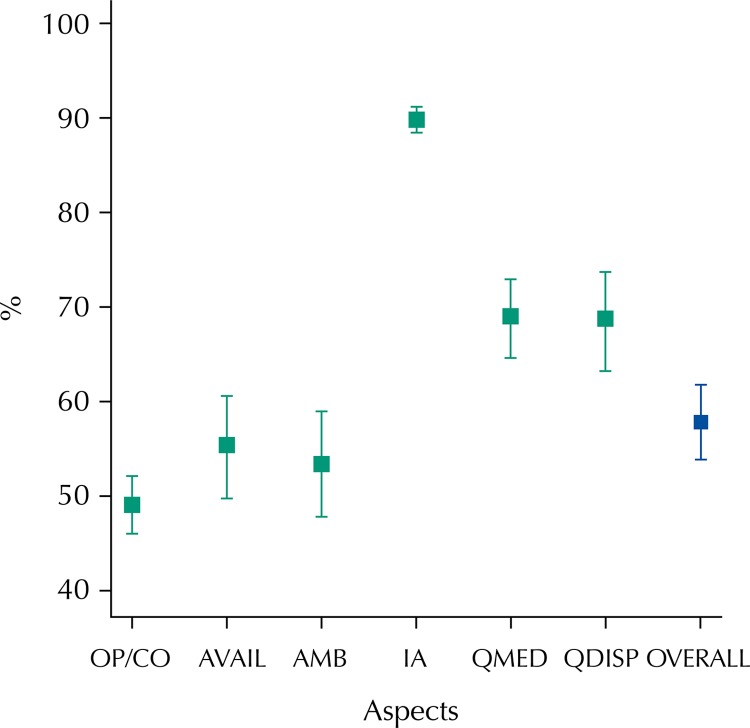
Proportion of patients satisfied with pharmaceutical services in primary health care according to the aspects and sub-aspects. National Survey on Access, Use and Promotion of Rational Use of Medicines – Services, 2015.

OP/CO: opportunity/convenience; AVAIL: availability; AMB: ambiance; IA: interpersonal aspects; QMED: quality of medicines; QDISP: quality of dispensation; OVERALL: overall satisfaction

Regarding ambiance, patients evaluated hygiene (90.9%) and comfort (74.2%) as satisfactory. Regarding the quality of medicines, the vast majority (87%) evaluated that the medicine was properly working for their disease and considered the medicines obtained in pharmacies of the SUS equal to or better than medicines bought at other pharmacies (93.2%).

As for dispensation, 78.7% of patients reported that they received information about the use of medicines and 94.8% said they understood the information. As for interpersonal aspects, patients have evaluated the service (90.5%) and the respectful and polite service (93.1%) as satisfactory (data not presented in table).

Regarding overall patient satisfaction, only the variables sex, age range, limitation due to diseases, and self-perception of health remained in the final multiple logistic model.

The following aspects were significantly associated with the highest overall patient satisfaction: being a male; being 30 years old or more, compared to the 18–29 age group; living in the Southeast region, compared to residents of the Midwest region; considering his own health very good or good, compared to those who consider it very bad; needing a maximum of one emergency service in the past 12 months, compared to those who needed two or more; and not feeling pain and malaise (Tables 1 and 2).

**Table 1:** Multiple logistic model of patient satisfaction with pharmaceutical services in the primary health care regarding the aspects, sub-aspects, and overall satisfaction, according to sociodemographic characteristics. National Survey on Access, Use and Promotion of Rational Use of Medicines – Services, 2015.

Variable	Opportunity/Convenience (n = 4,441)			Availability (n = 4,620)			Quality of medicines (n = 4,886)			Ambiance (n = 2,852)			Interpersonal aspects (n = 5,060)			Overall satisfaction (n = 4,768)		
					
	OR^a^	95%CI	p	OR^a^	95%CI	p	OR^a^	95%CI	p	OR^a^	95%CI	p	OR^a^	95%CI	p	OR^a^	95%CI	p
Sex																		
Male	1.165	(1.013–1.339)	0.032	1.269	(1.081–1.488)	0.003				1.190	(1.028–1.378)				0.022	1.261	(1.087–1.464)	0.002
Female	1	-	-	1	-	-	-	-	-	-	-	-	1	-	-	1	-	-
Age group (years)							-	-	-	-								
18 to 29	1	-	-	-	-	-	1	-	-	1	-	-	1	-	-	1	-	-
30 to 49	1.054	(0.889–1.250)	0.543	-	-	-	1.173	(0.975–1.412)	0.091	1.105	(0.921–1.325)	0.283	1.263	(0.933–1.708)	0.13	1.215	(1.007–1.466)	0.043
50 to 69	1.602	(1.288–1.993)	< 0.001	-	-	-	1.562	(1.288–1.893)	< 0.001	1.553	(1.206–1.999)	0.001	1.645	(1.098–2.463)	0.016	1.766	(1.387–2.248)	< 0.001
70 or more	1.994	(1.494–2.661)	< 0.001	-	-	-	2.275	(1.584–3.269)	< 0.001	1.911	(1.371–2.664)	< 0.001	2.081	(1.085–3.994)	0.028	2.222	(1.628–3.031)	< 0.001
Skin color																		
Nonwhite	-	-	-	1	-	-	-	-	-	-	-	-	-	-	-	-	-	-
White	-	-	-	1.238	(1.041–1.472)	0.016	-	-	-	-	-	-	-	-	-	-	-	-
Education level																		
Illiterate	1	-	-	1	-	-	-	-	-	1.568	(1.005–2.447)	0.048	2.156	(1.208–3.848)	0.01	-	-	-
Elementary school	1.098	(0.811–1.488)	0.545	1.851	(1.364–2.513)	< 0.001	-	-	-	1.697	(1.203–2.393)		2.116	(1.407–3.182)	< 0.001	-	-	-
High School	1.206	(0.861–1.691)	0.277	1.962	(1.501–2.566)	< 0.001	-	-	-	1.217	(0.849–1.744)	0.285	1.4	(0.879–2.230)	0.157	-	-	-
Higher Education	1.365	(0.931–2.003)	0.112	1.737	(1.187–2.542)	0.005	-	-	-	1	-	-	1	-	-	-	-	-
ABEP classification^b^																		
A or B	-	-	-	-	-	-	1	-	-	-	-	-	-	-	-	-	-	-
C	-	-	-	-	-	-	1.243	(1.042–2.483)	0.016	-	-	-	-	-	-	-	-	-
D or E	-	-	-	-	-	-	1.473	(1.170–1.854)	0.001	-	-	-	-	-	-	-	-	-
Region of residence																		
Midwest	1	-	-	1	-	-	1	-	-	1	-	-	1	-	-	1	-	-
North	0.768	(0.532–1.109)	0.160	1.341	(0.759–2.370)	0.312	1.315	(0.926–1.867)	0.126	1.05	(0.600–1.836)	0.865	0.884	(0.563–1.390)	0.594	1.255	(0.947–1.662)	0.114
Northeast	1.118	(0.782–1.600)	0.541	1.882	(1.091–3.247)	0.023	2.049	(1.364–3.080)	0.002	1.291	(0.779–2.142)	0.321	0.998	(0.640–1.555)	0.993	1.209	(0.805–1.815)	0.360
South	0.994	(0.760–1.301)	0.968	1.484	(0.837–2.633)	0.177	1.610	(1.000–2.592)	0.050	1.373	(0.871–2.167)	0.172	1.067	(0.707–1.607)	0.758	1.155	(0.788–1.691)	0.461
Southeast	0.990	(0.744–1.317)	0.946	1.945	(1.133–3.339)	0.016	1.549	(1.025–2.340)	0.038	2.066	(1.289–3.312)		1.422	(0.945–2.140)	0.091	1.596	(1.060–2.402)	0.025

^a^ Significant variables at the 5% level after adjusted analysis.

^b^ Brazil Economic Classification Criteria – Brazilian Market Research Association – ABEP 2013 – www.abep.org

Source: PNAUM – Services, 2015.

**Table 2:** Multiple logistic model of patient satisfaction with pharmaceutical services in the primary health care regarding the aspects, sub-aspects, and overall satisfaction, according to health and service use characteristics. PNAUM – Services, Brazil 2015.

Variable	Opportunity/Convenience (n = 4,441)			Availability (n = 4,620)			Quality of medicines (n = 4,886)			Ambiance (n = 2,852)			Interpersonal aspects (n = 5,060)			Overall satisfaction (n = 4,768)		
					
	OR*	95%CI	p	OR*	95%CI	p	OR*	95%CI	p	OR*	95%CI	p	OR*	95%CI	p	OR*	95%CI	p
Number of emergency health services in the past 12 months*																		
None	-	-	-	1.887	(1.487–2.395)	< 0.001	1.489	(1.130–1.961)	0.028	1.896	(1.437–2.502)	< 0.001	2.551	(1.781–3.652)	< 0.001	1.801	(1.383–2.346)	< 0.001
One	-	-	-	1.453	(1.124–1.879)	0.004	1.145	(0.908–1.444)	0.253	1.700	(1.220–2.370)	0.002	2.003	(1.346–2.981)	< 0.001	1.657		< 0.001
Two or more	-	-	-	1	-	-	1	-	-	1	-	-	1	-	-	1	-	-
Self-perception of health*																		
Very good	2.051	(1.235–3.405)	0.006	1.820	(0.917–3.612)	0.087	2.027	(1.080–3.803)	0.028	-	-	-	-	-	-	2.036	(1.163–3.564)	0.014
Good	1.697	(1.035–2.782)	0.036	2.288	(1.221–4.287)	0.010	1.486	(0.890–2.481)	0.130	-	-	-	-	-	-	1.729	(1.037–2.883)	0.036
Regular	1.246	(0.776–2.000)	0.364	1.727	(0.949–3.141)	0.074	1.079	(0.633–1.838)	0.781	-	-	-	-	-	-	1.294	(0.783–2.138)	0.315
Bad	1.152	(0.696–1.907)	0.581	1.257	(0.659–2.398)	0.487	0.981	(0.587–1.641)	0.943	-	-	-	-	-	-	1.048	(0.583–1.887)	0.875
Very bad	1	-	-	1	-	-	1	-	-	-	-	-	-	-	-	1	-	-
Limitations due to disease*																		
No	1.278	(1.092–1.495)	0.002	-	-	-	-	-	-	-	-	-	-	-	-	-	-	-
Yes	1	-	-	-	-	-	-	-	-	-	-	-	-	-	-	-	-	-
Pain and malaise*																		
No	-	-	-	1.496	(1.232–1.818)	< 0.001	1.441	(1.224–1.697)	< 0.001	1.574	(1.288–1.924)	< 0.001	1.628	(1.238–2.141)	< 0.001	1.486	(1.271–1.736)	< 0.001
Yes	-	-	-	1	-	-	1	-	-	1	-	-	1	-	-	1	-	-
Anxiety and depression*																		
No	-	-	-	-	-	-	1.212	(1.025–1.432)	0.025	-	-	-	1.352	(1.053–1.736)	0.018	-	-	-
Yes	-	-	-	-	-	-	1			-	-	-	1	-	-	-	-	-
Number of medicines																		
0	-	-	-	2.660	(1.678–4.216)	< 0.001	1.262	(0.849–1.876)	0.250	-	-	-	-	-	-	-	-	-
1	-	-	-	2.360	(1.602–3.478)	< 0.001	1.614	(1.144–2.277)	0.007	-	-	-	-	-	-	-	-	-
2	-	-	-	1.676	(1.192–2.358)		1.174	(0.856–1.610)	0.319	-	-	-	-	-	-	-	-	-
3	-	-	-	1.450	(1.016–2.070)	0.041	1.031	(0.764–1.393)	0.841	-	-	-	-	-	-	-	-	-
4	-	-	-	1.452	(1.009–2.088)	0.045	0.964	(0.695–1.337)	0.824	-	-	-	-	-	-	-	-	-
5 or more	-	-	-	1	-	-	1	-	-	-	-	-	-	-	-	-	-	-

* Significant variables at the 5% level after adjusted analysis.

Source: PNAUM – Services, 2015.

In the sub-aspect of technical quality of dispensation, it was not possible to obtain a multiple model, because despite variables significance, there was not a combination that would generate a proper adjustment. Thus, the evaluation of satisfaction in this sub-aspect was carried out using only a bivariate analysis and significantly associated with sex, marital status, skin color, education, patient of SUS, number of medical consultations in the past 12 months, and anxiety and depression (data not presented in table).

## DISCUSSION

Most interviewed patients were satisfied with pharmaceutical services in primary health care in the cities. Satisfaction with the service was a relevant factor in the overall patient satisfaction.

Literature shows that interpersonal quality of care is a determinant factor for patient satisfaction^5^. Opportunity/convenience had the smallest satisfaction level, which suggests the necessity of rethinking the structure where the services are provided. This will contribute even more to increase satisfaction with ambiance and with items regarding patient safety.

Studies showed an association between continuous care and patient satisfaction^7^ ^,^ ^18^. The degree of satisfaction found may be explained by the structure of SUS, which promotes continuous care, especially to patients with chronic conditions, such as hypertension and diabetes.

The degree of satisfaction of most patients for pharmaceutical services in primary health care suggests that implemented phamaceutical policies have made possible the creation of a capillary service network at a local sphere to offer these services to the population who attend the SUS.

According to analysis carried out by IRT, a methodological strategy that considered each item particularly without revealing total scores, the conclusions do not depend exclusively on the questionnaire, but on each item. IRT enables a new proposal for statistical analysis centered on each item, a perspective that transcends the limitations of classical theory, in which the model for scale construction is based directly on the result obtained from the tool as a whole^3^.

One of the objectives of this study was to measure the degree of patient satisfaction with the services. The vast majority of the questions present closed-ended questions to facilitate analysis, however, it presents limitations on the understanding of how patients perceive themselves in relation to the health system. It doesn’t contemplate beliefs, ways of life, conceptions of the health-disease process, and each patient’s expectations, which certainly affect the way these services are used and evaluated^22^.

Regarding sex, most respondents are female, which alludes to the belief that self-care and care for others are values associated with femininity, linked to the lower labor force participation rate of women in our society^14^. However, satisfaction is strongly associated with men, which is consistent with the literature that shows services receiving more criticism from women, who are thus good informants for surveys^11^.

Patients 30 years or older are more satisfied with pharmaceutical services, which corroborates the findings in the literature according to which older age groups have less expectations in relation to services^12^.

Regarding the region of the Country, we observed that patients from the Southeast region were more satisfied with pharmaceutical services than patients from other regions. Such data suggests that in more developed regions, such as the Southeast and South regions, the primary health network would be more organized, as well as provides a better offer of health services and, in this case, pharmaceutical services^8^.

As expected, the patient’s current state of health may potentially affect the degree of patient satisfaction with health services and, in the case of this study, pharmaceutical services, not only by the severity of case, but also by physical, psychological, social, or mental limitations due to disease^12^.

This can help to explain how patient satisfaction is associated with those who received up to one emergency service, compared to those who received two or more. Likewise, in health self-perception, patient satisfaction is associated with those who consider their health very good and good, compared to those who consider their health regular, bad, or very bad. Regarding the state of health, it is associated with patient satisfaction, among those who do not feel pain and discomfort to the detriment of those who feel it.

In summary, satisfaction with services is not itself a measure of health care quality. It may be, however, indirectly associated with quality, since it can influence the search for determined kinds of services, which affect the state of health^21^.

Therefore, for a better understanding of the results, it is necessary to identify and analyze associated factors, not considered in this survey, e.g., sociodemographic, behavioral and cultural differences that affect patient satisfaction^5,9.^

In this sense, the patient satisfaction survey brings health services and the community closer, because it increases awareness about these patients’ needs^23^.

Another limitation of this study is related to the scarcity of publications about the evaluation and satisfaction with pharmaceutical services in the SUS, which made it difficult to establish comparisons among the results obtained.

Patient satisfaction with pharmaceutical services in primary health care was satisfactory. Differences found among evaluated dimensions, however, show gaps in the quality of services offered. In Brazil, the assessment of pharmaceutical services is in its early stages, which shows the necessity of making PNAUM the baseline to evaluate whether pharmaceutical services meet the patient’s needs. It is expected that the results obtained would foster actions directed to the development of pharmaceutical services in the country.

## Footnotes

a Associação Brasileira de Empresas de Pesquisa. Critério de classificação econômica Brasil: alterações na aplicação do Critério Brasil, válida a partir de 01/01/2014. São Paulo: ABEP; 2014 [cited 2016 Mar 1]. Available from: www.abep.org/Servicos/Download.aspx?id=01
